# Supplementary material for: Aerogel Perfusion-Prepared h-BN/CNF Composite Film with Multiple Thermally Conductive Pathways and High Thermal Conductivity
Source: Nanomaterials (Basel). 2019 Jul 23;9(7):1051. doi: 10.3390/nano9071051 (PMC6669481; doi:10.3390/nano9071051)
Supplement: Supplementary file 1 [file nanomaterials-09-01051-s001.pdf]

Supporting Information

Aerogel Perfusion-Prepared h-BN/CNF Composite Film with Multiple Thermally Conductive Pathways and High Thermal Conductivity

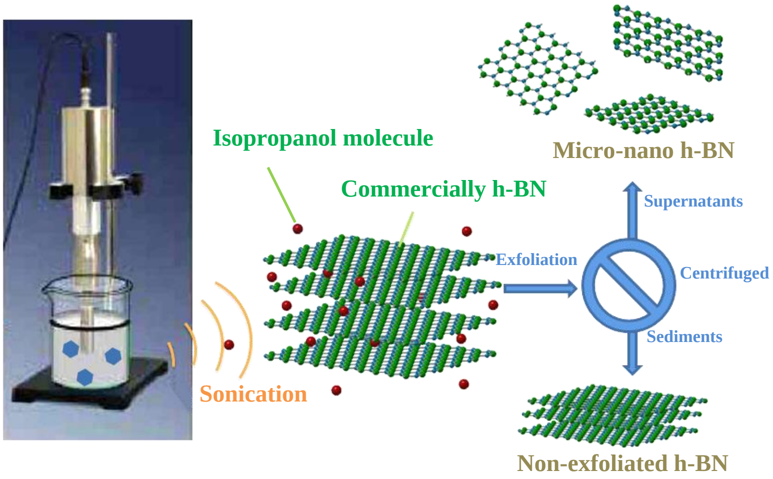

Figure S1. Schematic of exfoliation of h-BN.

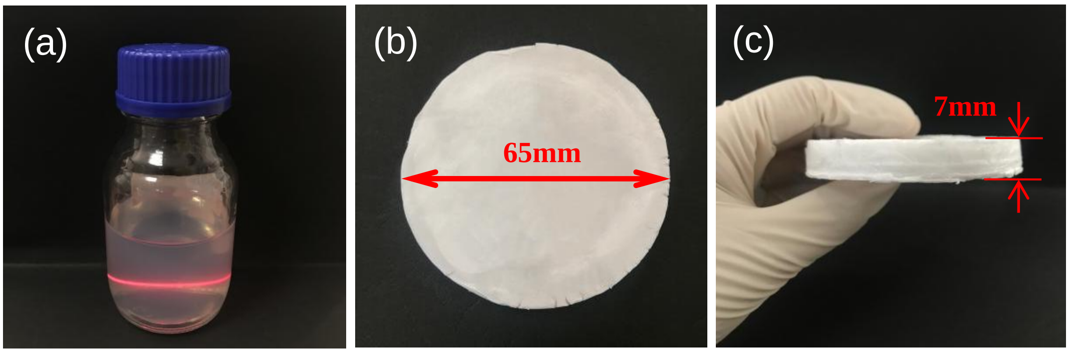

Figure S2. (**a**) Tyndall phenomenon of CNF. (**b**) and (**c**) Optical photographs of CNF aerogel, red arrows indicated the diameter and the thickness of the CNF aerogel were 65 mm and 7mm.

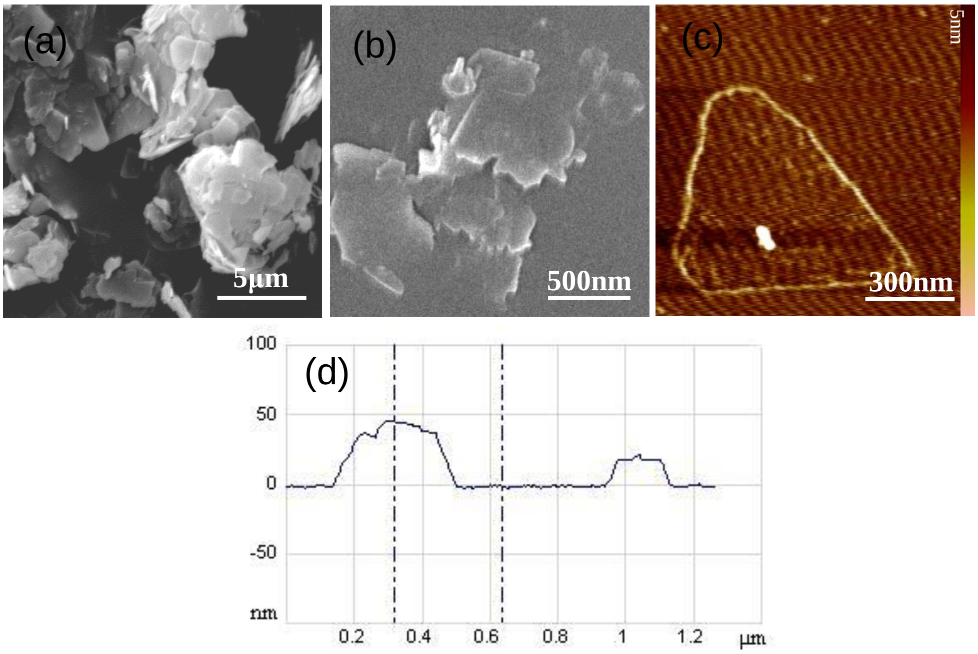

Figure S3. (**a**) SEM image of commercially h-BN. (**b**) SEM image of exfoliated h-BN. (**c**) AFM topographic image of exfoliated h-BN. (**d**) The corresponding height distribution measured from AFM topographic data from Figure 2f.

Table S1. Comparing the specific heat, thermal diffusivity and density of h-BN /CNF composite films prepared by different methods (25 °C).

h-BN	Blended composite film			Aerogel perfusion composite film		
Content	Specific Heat	Thermal Diffusivity	Density	Specific Heat	Thermal Diffusivity	Density
（wt%）	$J \cdot g^{-1}k^{-1}$	$mm^2 \cdot s^{-1}$	$g \cdot cm^{-3}$	$J \cdot g^{-1}k^{-1}$	$mm^2 \cdot s^{-1}$	$g \cdot cm^{-3}$
0	1.489 ± 0.001	0.268 ± 0.003	1.035 ± 0.002	1.488 ± 0.002	0.268 ± 0.002	1.036 ± 0.002
4.79	1.446 ± 0.003	0.270 ± 0.003	1.076 ± 0.001	1.450 ± 0.003	0.314 ± 0.003	1.088 ± 0.002
9.09	1.414 ±0.002	0.277 ± 0.001	1.116 ± 0.001	1.411 ± 0.001	0.566 ± 0.001	1.119 ± 0.001
13.07	1.378 ± 0.003	0.302 ± 0.001	1.156 ± 0.002	1.377 ± 0.001	0.664 ± 0.002	1.165 ± 0.003
16.67	1.354 ± 0.001	0.340 ± 0.002	1.193 ± 0.001	1.355 ± 0.003	0.785 ± 0.001	1.197 ± 0.002
20.02	1.289 ± 0.003	0.387 ± 0.002	1.233 ± 0.002	1.284 ± 0.004	0.882 ± 0.003	1.257± 0.001
23.08	1.248 ± 0.004	0.425 + 0.003	1.278 ± 0.002	1.239 ± 0.002	0.931 ± 0.001	1.289 ± 0.001
